# The estimation of crop emergence in potatoes by UAV RGB imagery

**DOI:** 10.1186/s13007-019-0399-7

**Published:** 2019-02-12

**Authors:** Bo Li, Xiangming Xu, Jiwan Han, Li Zhang, Chunsong Bian, Liping Jin, Jiangang Liu

**Affiliations:** 1 Institute of Vegetables and Flowers, Chinese Academy of Agricultural Sciences (CAAS)/Key Laboratory of Biology and Genetic Improvement of Tuber and Root Crops, Ministry of Agriculture, Beijing, China; 2 NIAB EMR, New Road, East Malling, Kent, ME19 4BD UK; 3 Institute of Biological, Environmental and Rural Sciences (IBERS), Aberystwyth University, Penglais, Aberystwyth, Ceredigion, SY23 3FL UK

**Keywords:** Unmanned aerial vehicle (UAV), Potato, Image analysis, Remote sensing, Crop emergence, Random Forest

## Abstract

**Background:**

Crop emergence and canopy cover are important physiological traits for potato (*Solanum tuberosum* L.) cultivar evaluation and nutrients management. They play important roles in variety screening, field management and yield prediction. Traditional manual assessment of these traits is not only laborious but often subjective.

**Results:**

In this study, semi-automated image analysis software was developed to estimate crop emergence from high-resolution RGB ortho-images captured from an unmanned aerial vehicle (UAV). Potato plant objects were extracted from bare soil using Excess Green Index and Otsu thresholding methods. Six morphological features were calculated from the images to be variables of a Random Forest classifier for estimating the number of potato plants at emergence stage. The outputs were then used to estimate crop emergence in three field experiments that were designed to investigate the effects of cultivars, levels of potassium (K) fertiliser input, and new compound fertilisers on potato growth. The results indicated that RGB UAV image analysis can accurately estimate potato crop emergence rate in comparison to manual assessment, with correlation coefficient ($r^{2}$) of 0.96 and provide an efficient tool to evaluate emergence uniformity.

**Conclusions:**

The proposed UAV image analysis method is a promising tool for use as a high throughput phenotyping method for assessing potato crop development at emergence stage. It can also facilitate future studies on optimizing fertiliser management and improving emergence consistency.

## Background

Potato is one of the most important economic crops in the world with an annual production of more than 380 million tons [1]. Its yield is affected by many factors including cultivars and nutrient supplies [2]. China is the world’s largest potato producer and consumer, having 5.8 million hectares of cultivated area. In order to improve potato yield, the utilization of resources such as chemical fertilisers and pesticides has increased steadily, leading to potential economic waste and environmental pollution [3]. Optimization of nutrient management strategies is required for all commercial potato cultivars in China.

Potato emergence dynamics, including emergence rate and uniformity, play important roles in screening varieties [4], field management [5, 6] and yield prediction [5], and can be affected by many factors, such as seed potato quality, dormancy period, soil temperature, water stress and nutrient deficiency [7, 8]. Consistent emergence is always desirable as it leads to more efficient crop management. Crop canopy cover directly determines the amount of sunlight interception and hence affects photosynthetic efficiency. It is one of the most frequently-used traits for estimating canopy structure with remote sensing technology at the early growth stage [6]. The measurement of emergence rate and uniformity is crucially important for field-scale phenotyping, especially in crop breeding and precision agronomy. Traditionally, crop emergence is assessed via time-consuming manual counting; whereas canopy cover is estimated by subjective and inaccurate manual scoring. The repeatability of both manual assessment methods can be low, especially when they are used in large agronomy and breeding experiments, which may have thousands of trial plots [9].

Potassium (K) is an activator of a plethora of enzymes, notably with roles including carbohydrate, nucleic acid and protein synthesis, protein operation and soluble sugar transport. Application of K fertilisers can increase leaf chlorophyll content and photosynthesis rate. Understanding the effect of K fertilisers at the early stage of potato growth is crucial for developing resource input strategies. Excessive application of synthetic fertilisers can result in serious environmental pollution. Through the application of novel products, such as bio-organic and microbial fertilisers, the use of synthetic fertilisers can be reduced.

Unmanned aerial vehicle (UAV) imaging is a rapidly growing technology in recent years and has been widely applied in crop monitoring due to its high efficiency, high spatial and temporal resolution, low cost and easy customization [10]. Since 2010, high-throughput phenotyping by UAV imaging has been introduced to precision agriculture [11] in a range of applications, such as the detection of abiotic stress [12], nutrient deficiency [13] and biotic stress [14, 15], weed management [16], plant growth monitoring [17–19] and yield prediction [20, 21]. In previous studies, low-altitude UAV equipped with a RGB camera was shown to be an effective method for counting rapeseed seedlings [22] and estimating canopy cover of cotton, sorghum and sugarcane [9, 23]. Using UAV imaging to estimate potato crop emergence has only been reported once recently [24]. By using a multispectral camera, image-based plant counting showed a satisfactory correlation with manual counting. But canopy cover and emergence uniformity were not reported. UAV equipped with a RGB imaging sensor has been applied to count wheat [25] and rapeseed seedlings [22] at the emergence stage. Both studies applied an improved vegetation index, i.e. Excess Green minus Excess Red (ExG-ExR) [26] to separate green canopy from soil. Several morphological features were calculated and modelled using Support Vector Machine (SVM) and Multiple Linear Regression (MLR) for estimating seedling number.

In this study, we developed a robust Random Forest based UAV image analysis software to calculate emergence rate, estimate crop canopy cover and assess the emergence uniformity. This image analysis system were then applied to data from three large field experiments that evaluated the accuracy in estimating crop emergence against manual assessment, and objectively investigated the differences in crop emergence rates and uniformity under various cultivars and nutrient input regimes.

## Methods

### Study site

**Fig. 1:**
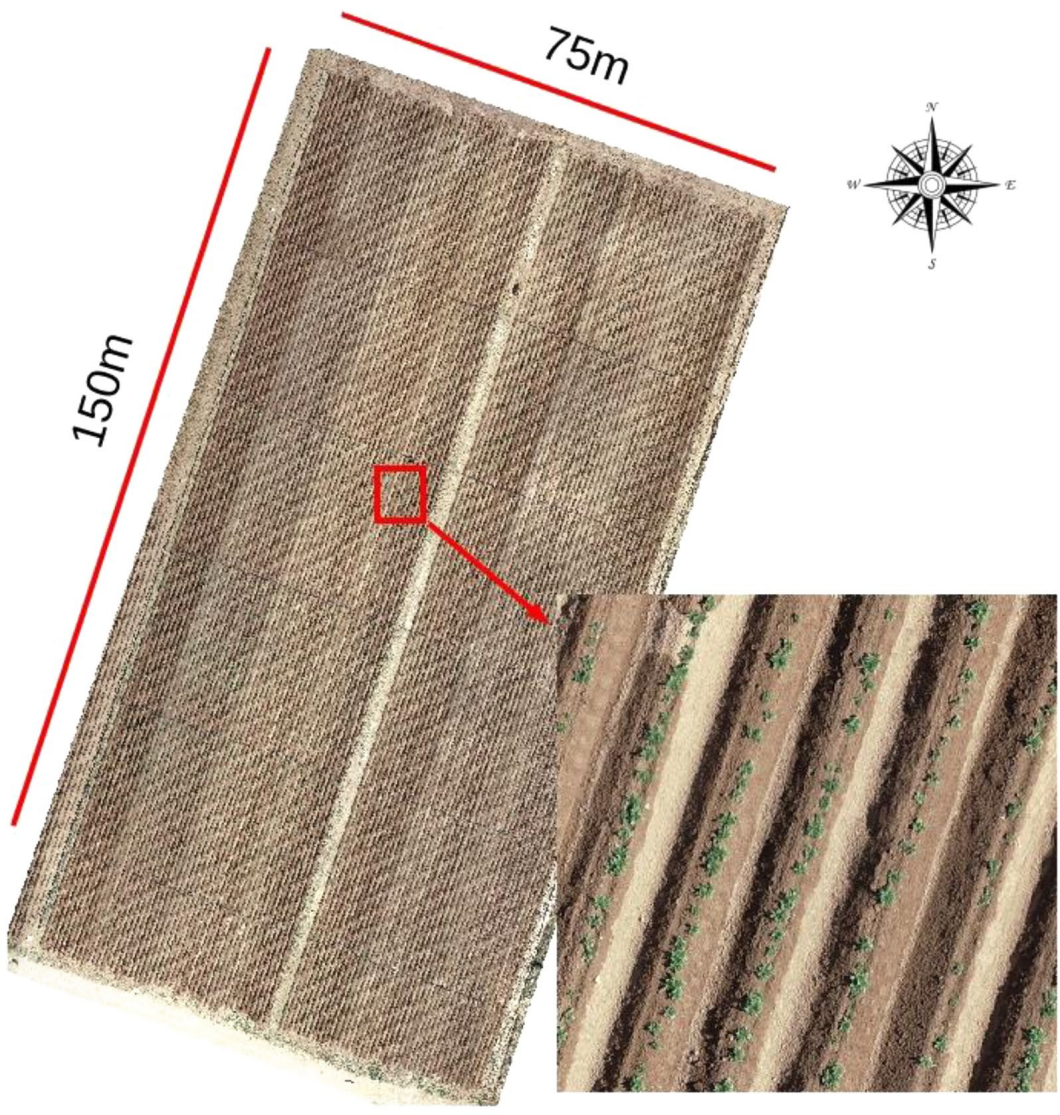
The field map of the experiment

The study site shown in Fig. 1 is located in a research station of the Chinese Academy of Agricultural Sciences (CAAS) in Zhangjiakou, Hebei, China (41°28′28.82″N, 115°03′43.91″E, altitude 1390 m). This area is in the mid temperate zone with Chestnut soil type and monsoon climate, where the annual precipitation and sunshine hours are around 300 mm and 2898 h, respectively. Three field experiments were designed to assess the impacts of cultivars and nutrient inputs on potato emergence and subsequent growth. Seed potatoes were sown on 6th May, 2018. A selective herbicide (DuPont Matrix) was applied at the emergence stage. Among the six cultivars used in the three experiments, ‘Favorita’, ‘Zhongshu 5’ and ‘Zhongshu 10’ are early maturing cultivars, whilst ‘Zhongshu 18’, ‘Zhongshu 19’ and ‘Shepody’ are late maturing cultivars.

Experiment 1 contained two sub-experiments, focusing on the comparison of two pairs of cultivars: (1) ‘Favorita’ versus ‘Zhongshu 19’, and (2) ‘Zhongshu 10’ versus ‘Zhongshu 18’. Each pair of cultivars formed a sub-experiment. The experimental plots were designed in blocks, laying towards 2 directions. One direction had five blocks, each with one level of N fertiliser input (0, 40, 80, 120 and 160 kg ha^−1^). Along the other direction, there were three blocks with the same cultivar pairs. In total, the trial site was divided into 30 plots, 15 for each sub-experiment. Within each plot there were two subplots, each allocated to one of the two paired cultivars. The size of each subplot was 8 × 5.3 m, containing six rows with an evenly sowed 270 seeds in total.

Experiment 2 was designed to study the effects of K fertiliser inputs on the emergence of two selected late maturing cultivars. This experimental plot was also laid in blocks in two directions. In one direction, there were two blocks, each allocated to one of the two late maturing cultivars (‘Zhongshu 18’ and ‘Shepody’). Along the other direction, there were three blocks. In total, this experiment had six plots. Within each plot there were four subplots, each allocated to one of the four K input levels: 0, 75, 150 and 225 kg ha^−1^. The subplot size and sowing date were the same as in Experiment 1.

Experiment 3 investigated the effect of eight mixed fertiliser treatments (Table 1) on potato emergence and subsequent crop development. Similarly, this experimental plot was designed in blocks in two directions. In one direction, there were two blocks, where ‘Zhongshu 5’ and ‘Zhongshu 18’ were allocated. Along the other direction, there were three blocks. In other words, the experiment was divided into six plots. Each plot was divided into eight subplots, each allocated to one of the eight fertiliser treatments (Table 1). The subplot size was 6 × 5.3 m, having six rows with evenly sowed 210 seeds in total.

**Table 1:** Details of the eight compound fertiliser treatments used in Experiment 3 to study their effects on potato emergence and subsequent development

Treatment^a^	F1	F2	F3	F4
	Compound fertiliser (CF, kg ha^−1^) (N:P_2_O_5_:K_2_O =15:15:15)	F1 + Soil Conservation fertiliser (SCF, kg ha^−1^)	F1 + Soil Conservation fertiliser (SCF, kg ha^−1^)	F1 + Organic–inorganic fertiliser (OIF, kg ha^−1^)
Base fertiliser	CF300	CF300 + SCF300	CF300 + SCF150	CF300 + OIF300
Treatment^a^	F5	F6	F7	F8
	F1 + Organic–inorganic fertiliser (OIF, kg ha^−1^)	F1 + Compound microorganism (CM, kg ha^−1^)	F1 + Compound microorganism (CM, kg ha^−1^)	F1 + 25%F1
Base fertiliser	CF300 + OIF150	CF300 + CM80	CF300 + CM160	CF600

^a^CF: Sino-Arab Chemical Fertilisers Co. Ltd. (SACF), N:P_2_O_5_:K_2_O = 15:15:15; SCF: Guizhou Bao Tu Ecological Recycling Agriculture Technology Co. Ltd., N:P:K = 6:4:10; OIF: Yunnan Tumama Fertilisers Co., Ltd, N:P_2_O_5_:K_2_O = 8:8:14, Organic matter ≥ 12%; CM: *Bacillus subtilis*/*Bacillus licheniformis*, complex fermentation, microbial propagules ≥ 0.2 billion per gram

### UAV campaign

The field images were captured by a quadcopter lightweight UAV (DJI Phantom 4 Pro) 35 days after planting when some subplots showed > 50% emergence by visual assessment. The UAV was equipped with a high resolution RGB camera with a 1-inch CMOS sensor to generate 20 mega pixel images. A total of 473 images with a horizontal overlap of at least 60% were captured vertically at a 30 m flight height, showing a spatial resolution of ca. 0.5 cm. Pix4D software (Pix4D SA, Lausanne, Switzerland) was used to stitch all captured images together to generate a georeferenced orthomosaic image of the field.

### Image processing

A semi-automated image analysis software in C++ [27] with Open Computer Vision library (OpenCV 3.2) was developed, of which the graphic user interface (GUI) was designed by the Qt designer.

### Image segmentation

In order to target the canopy objects, it is significant to separate the regions of vegetation from the background. In previous studies, colour features converted from the original R, G and B channels have been used to enhance the contrast between green canopy and the background [28, 29], and a more sophisticated method employing machine learning model could perform the segmentation with 21 colour features [30]. For UAV remote sensing, the original RGB image was normally transformed into a greyscale image of a Vegetation Index (VI) [31, 33]. Two common VIs are Excessive Green Index (ExG) (Eq. 1) and Excess Green minus Excess Red Index (ExG-ExR) (Eq. 2). The latter one was successfully applied in counting wheat [25] and rapeseed seedling [22] at the emergence stage.

1 $$ {\text{ExG}}_{\text{x,y}} = 2{\text{G}}_{{{\text{x}},{\text{y}}}} - {\text{R}}_{{{\text{x}},{\text{y}}}} - {\text{B}}_{{{\text{x}},{\text{y}}}} $$

2 $$ \left( {{\text{ExG}} - {\text{ExR}}} \right)_{\text{x,y}} = 3{\text{G}}_{\text{x,y}} - 2.4{\text{R}}_{\text{x,y}} - {\text{B}}_{\text{x,y}} $$

where R_x,y_, G_x,y_ and B_x,y_, are defined as the reflectance intensities in red, green and blue bands of the images respectively, and x and y are the coordinate of a specific pixel.

To extract the target plant objects in the image, the Otsu thresholding method was applied to transform the VI greyscale image into a binary image with 0 assigned to pixels corresponding to the bare soil. The Otsu method performs automatic thresholding [32] and has been reported for the successful segmentation of green crops from the bare soil after applying ExG [33]. ExG-ExR converts the intensities of plant pixels to positive values and the background to negative values, therefore the arbitrary value 0 is used as the threshold. In this study, the accuracy of both plant extraction methods were measured using a quality factor (Q) defined by the Automatic Target Recognition Working Group (ATRWG) [26] as shown in Eq. 3:

3 $$ Q = \frac{{\mathop \sum \nolimits_{i,j = 0}^{i,j = w,h} (P_{i,j}  \;And\; B_{i,j} )}}{{\mathop \sum \nolimits_{i,j = 0}^{i,j = w,h} \left( {P_{i,j}  \;Or\; B_{i,j} } \right)}} $$

where w and h are the total number of rows and columns of the image, P is the binary image after plant segmentation using image analysis, B is the reference image generated by manual segmentation and i, j are the indices of row and column.

The quality factor Q was calculated from the images of the 15 selected subplots for comparison of the above two plant extraction methods. A perfect segmentation of plant is represented by Q as 1.0.

### Evaluation of crop emergence rate, crop emergence uniformity and average canopy cover

In the semi-automated image analysis software, the region of interest (ROI) within the selected plot was manually selected. Because the plot sizes were known, the ROI in both RGB and binary images can be cropped with a predefined width and height. With edge detection on the binary image, the closed contour could be obtained for each connected component that was corresponding to the plant object and a minimum area bounding box was fitted to each contour. Contours with less than 35 pixels were considered as noise and discarded. Due to the variability of plant growth, canopy overlapping was inevitable and therefore a bounding box might contain multiple plants.

It is difficult to separate each individual potato plant due to the complex overlapping, but the number of plants showed great influence on the morphology of plant object. Six morphological features (Table 2) were, therefore, calculated in this study to assess their correlation with plant number.

**Table 2:** The six morphological features calculated for each minimum area bounding box

Name	Meaning	Unit
Length	The length of the long side of the bounding box	cm
Length–width ratio	The ratio between the length and the width of the bounding box	Scalar
Area	Total area of the plant canopies within the bounding box	cm^2^
Perimeter	The perimeter of the connected contour of the canopy within the bounding box	cm
Convex area	Total area of the convex hull containing the connected contour of the canopy	cm^2^
Solidity	The ratio between the area of canopy and convex hull	Scalar

**Fig. 2:**
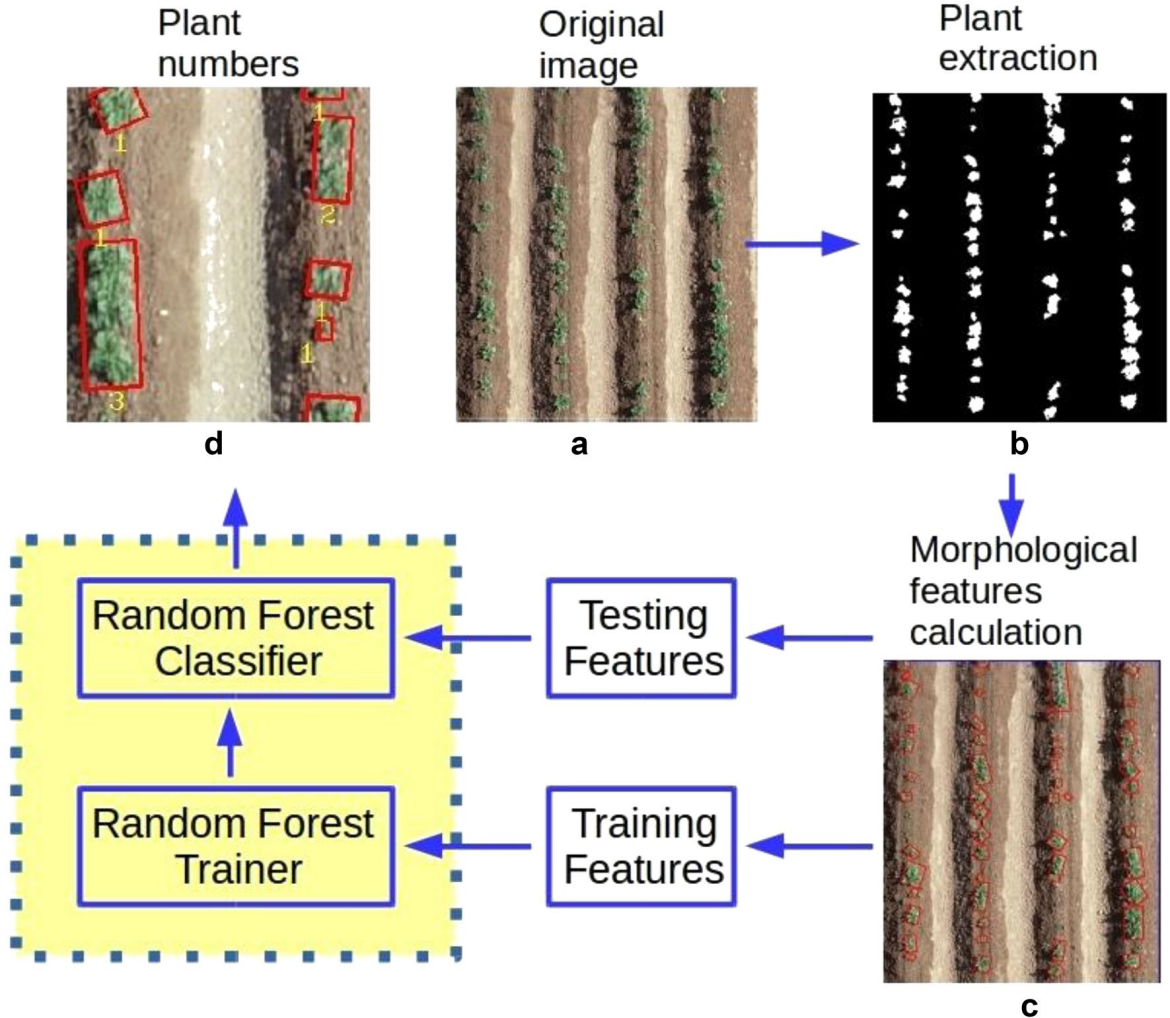
Flowchart of plant number estimation. **a** Original image; **b** binary image produced by the ExG + Otsu method; **c** connected contour detection, minimum area bounding box fitting and morphological features calculation. **d** Labelling estimated plant number below each minimum area bounding box

With morphological features, a Random Forest classifier [34] was applied for estimating plant number within a bounding box. Random Forest is a supervised classification method. A random forest classifier consists of a number of decision trees with each tree containing a random subset of variables, which can increase the diversity and robustness of the forest for prediction. The classifier predicts the class of a testing sample by taking the majority of unit votes cast from all individual trees [35]. Compared with other statistical modelling methods, Random Forest has a number of advantages. It does not need a variable pre-selection and is robust to variable noise. It is also more resistant to overfitting and capable of providing reliable error evaluation by out-of-bag data [36]. Moreover, Random Forest can rank the importance of variables. The importance score of a specific variable is calculated by averaging the difference of out-of-bag error after the permutation among all trees, and large score indicates greater importance [37]. For model calibration, 80% of the samples were split for training purpose and the remaining 20% of the samples referred to as out-of-bag were used for validation. In total, 540 plant objects across all treatments were selected from separate trial plots for the training data collection, and the numbers of plants within one plant object were labelled manually, ranging between 1 and 8. All proposed morphological features were calculated individually for each plant object and used as the variable vectors for Random Forest classifier. In general, more trees lead to more stable and robust prediction, but the improvement decreases with the number of trees increases and reaches constant at a certain point. The out-of-bag classification error of calibration remained constant after the number of trees reached 75, and the number 100 was set as the system default value as a reliable forest size. The flowchart of the whole process of counting estimation is given in Fig. 2.

The total canopy cover of a subplot was calculated by summing all pixels corresponding to potato canopy and then the average canopy cover per plant was calculated by dividing the total canopy cover by the number of estimated plants per subplot [38]. The variability in the canopy cover per plant within each subplot was then estimated based on the number of estimated plants. Within each subplot, the average canopy cover per plant was calculated for each minimum area bounding box, and the coefficient of variation (CV) was calculated based on all average values for the number of minimum bounding boxes.

### Data analysis

The emergence rate, average canopy cover and emergence uniformity were obtained for all subplots. The emergence rate calculated using UAV-based image analysis was correlated with the ground-truth data for all subplots. Coefficient of determination ($r^{2}$) and root mean square error (RMSE) were calculated. The effects of cultivars (Experiment 1), K fertiliser inputs (Experiment 2) and synthetic fertiliser replacement (Experiment 3) on these plant traits were assessed by the analysis of variance (ANOVA) using Genstat statistical package (version 13.0, VSN International Ltd. England). Once the overall effects for a given treatment factor were statistically significant, Fisher’s Least Significant Difference (LSD) test was used to compare pairs of treatments [39].

## Results

### Plant segmentation

**Fig. 3:**
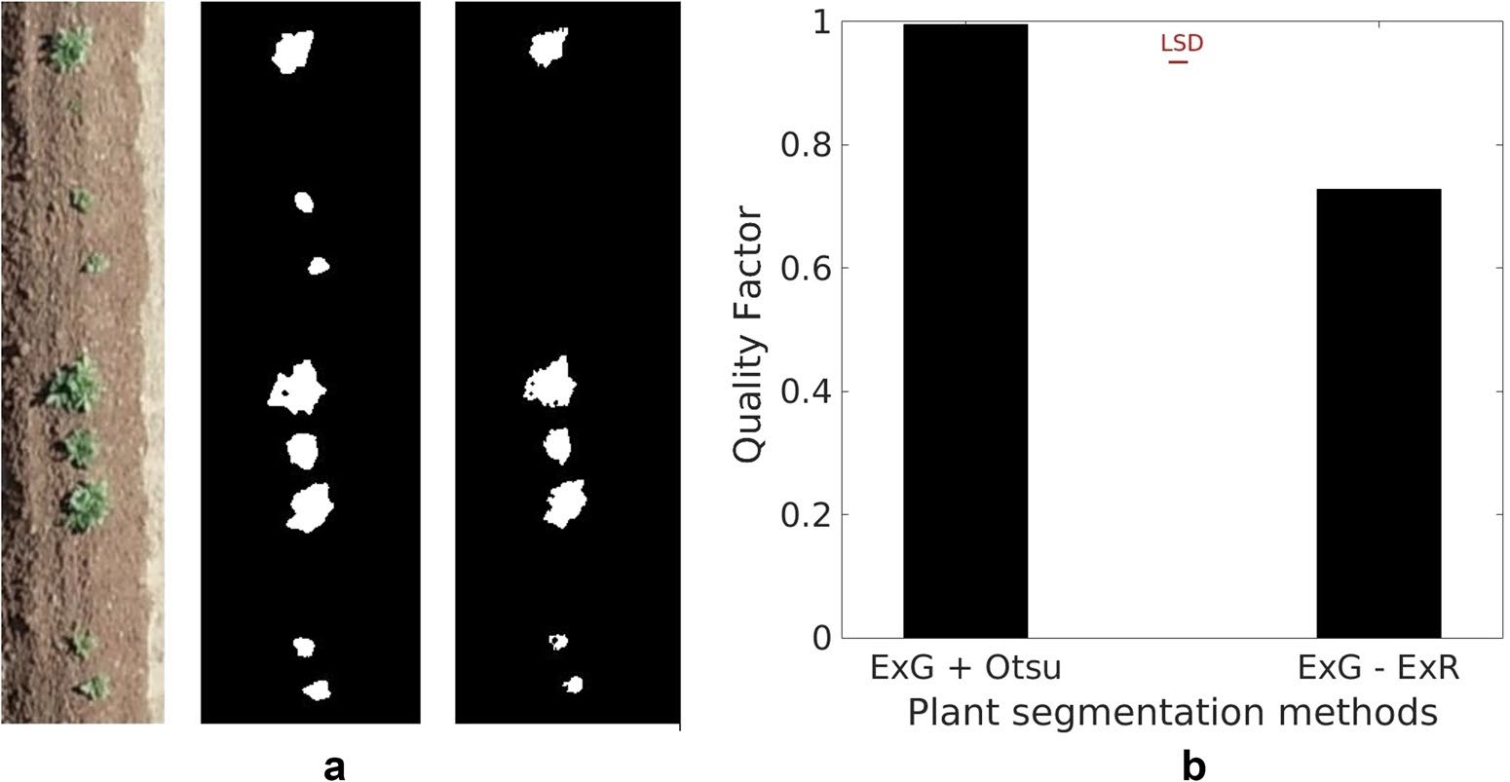
Original RGB image (**a)** left, plant extraction by ExG + Otsu (**a)** middle, plant extraction by ExG-ExR (**a)** right and comparison of quality factors of two methods (**b**)

Figure 3 compares the two plant segmentation methods used in this study. The ExG + Otsu method performed better than the ExG-ExR method, which missed a significant number of plant pixels and grouped them into the dark background. The Q value for the ExG-ExR method was significantly lower than the ExG + Otsu method (*F*_1,28_ = 180.0, *p* < 0.001). Consequently, the ExG + Otsu method was selected as the segmentation method in this study.

### Evaluation of the image analysis algorithm

**Fig. 4:**
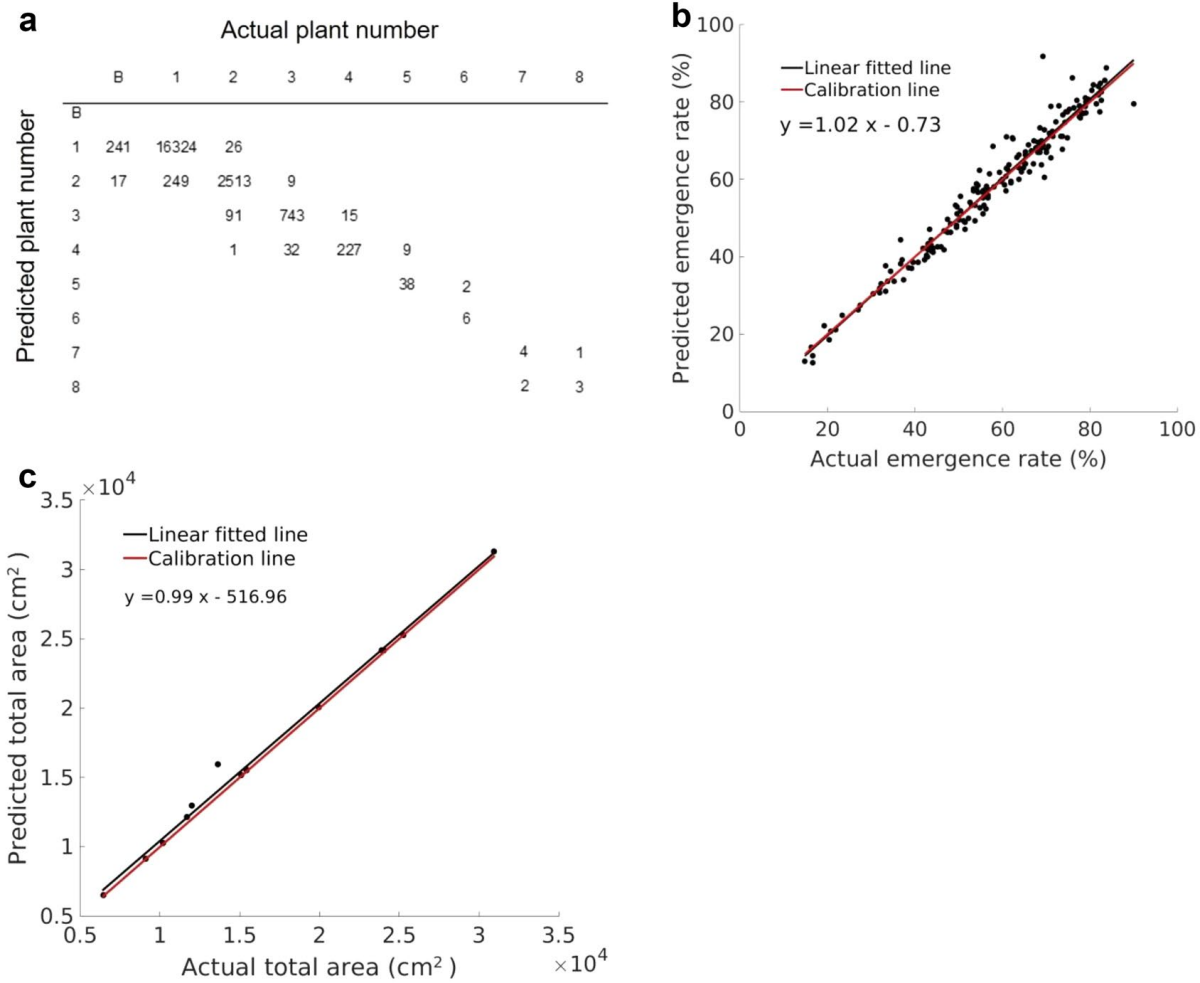
Confusion matrix of the actual and predicted plant numbers, B—Background (**a**). Comparison of the estimated potato crop emergence rate by the UAV-based image analysis software with the manual counting. Black and red lines are the fitted linear line and the ideal 1:1 line, respectively (**b**). Comparison of total area of selected plots between ExG + Otsu segmentation method and manual segmentation (**c**)

**Fig. 5:**
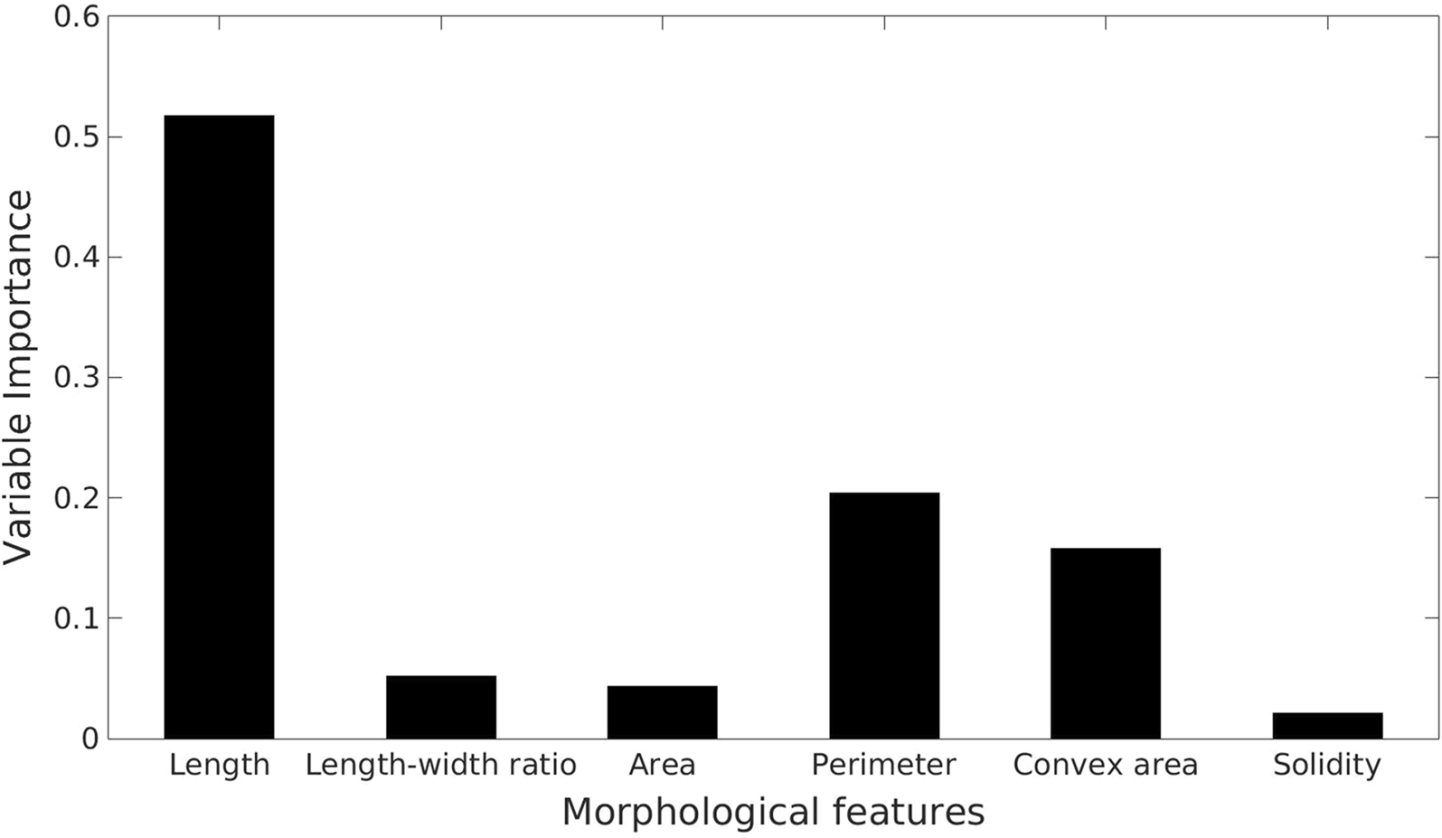
Variable importance of all morphological features

Large variations in the potato emergence rates were observed in the three experiments, ranging between 14.8 and 90.0%. Figure 4a shows the confusion matrix, comparing the actual and predicted number of plants within a plant object. The correct classification rate is 96.6%. However, when the actual plant number was two to four, overestimation was more likely to be found; on the other hand, underestimation was more likely for larger numbers of plants. As stated above, the emergence rate calculated by image analysis were compared with manual counting to determine the feasibility of the proposed method. Linear regression indicated a close agreement between UAV-based image analysis software and manual assessment with $r^{2}$ and RMSE at 0.96 and 3.63%, respectively (Fig. 4b). Relatively larger deviations were found between imaging and manual assessment for subplots showing larger emergence rates. This is likely due to the errors caused by greater canopy overlaps between plants and by possible presence of weeds. The importance of all morphological features in relation to plant number is given in Fig. 5. Not surprisingly, the length of the minimum area bounding box showed the highest value than all the other features as the potatoes were planted in rows. Perimeter and convex area were less important but significantly higher than the remaining features.

Similar comparison was also made to validate the area measurement between the proposed ExG + Otsu method and manual segmentation (Fig. 4c); there was a high correlation ($r^{2} = 0.99$) between the two measurements with RMSE at 617 cm^2^.

### Effect of cultivar and nutrient management on potato emergence

Following validation of estimating the emergence rate, the image analysis software was used for all experimental trial plots in order to validate this phenotyping method by quantifying the effect of cultivar, K fertiliser input and different organic–inorganic compound fertiliser applications on potato emergence through estimating emergence rate, uniformity and average canopy area.

### Emergence rate

**Fig. 6:**
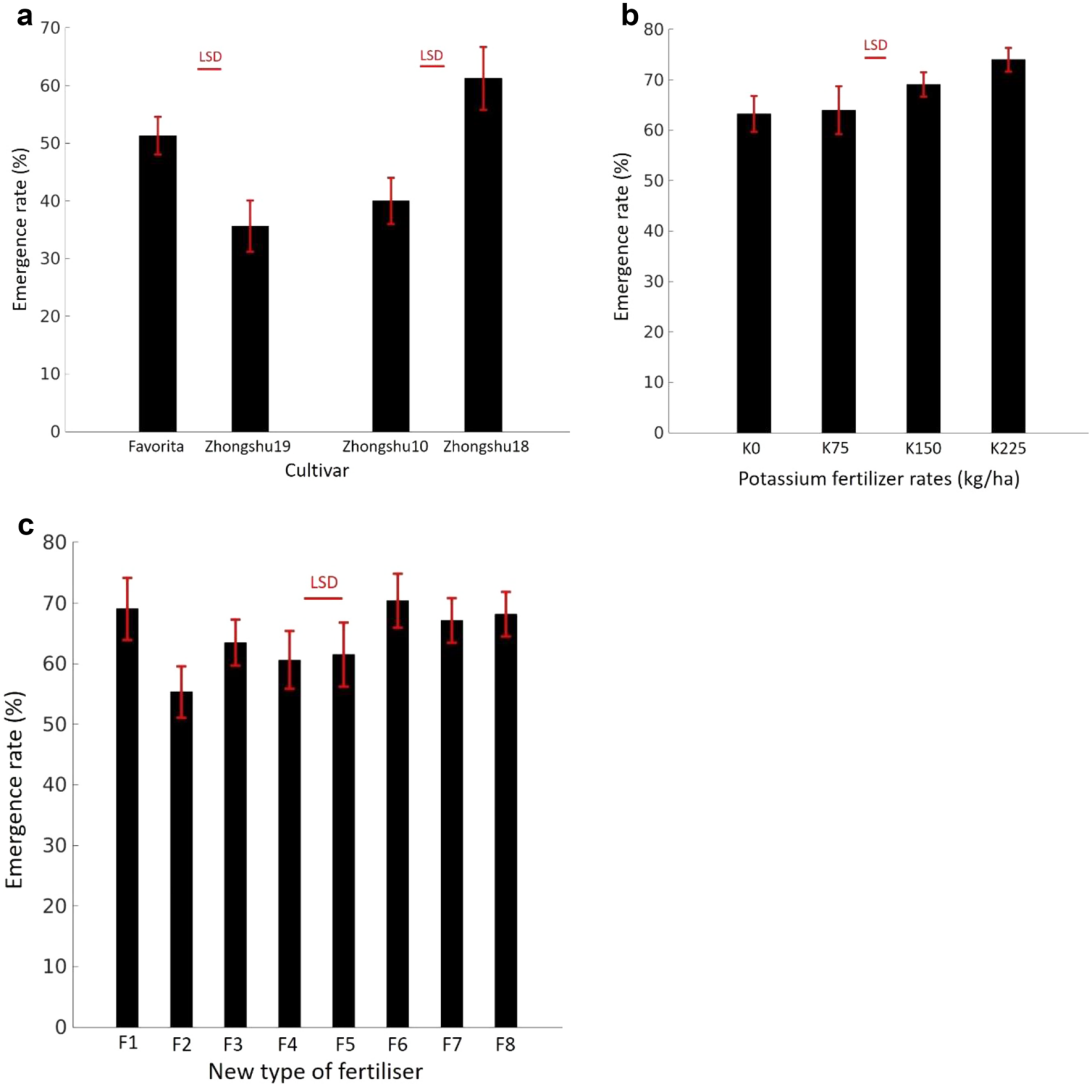
Estimated emergence rate of plants by the newly developed image analysis software for **a** multiple cultivars (Experiment 1), **b** different treatments of K fertiliser (Experiment 2), and **c** different organic–inorganic compound fertilizsr applications (Experiment 3). *LSD* least significant difference

ANOVA results showed significant differences in the potato emergence between the pairs of cultivars (Fig. 6a) in Experiment 1. Favorita showed a greater emergence rate than ‘Zhongshu 19’ (*F*_1,14_ = 20.75, *p* < 0.005). ‘Zhongshu 18’ showed a greater emergence rate than ‘Zhongshu 10’ (*F*_1,14_ = 28.90, *p* < 0.005).

Significant effects of K inputs on potato plant emergence (*F*_3,9_ = 6.57, *p* < 0.05) were observed in Experiment 2. Applying K fertiliser at 225 kg ha^−1^ led to a higher emergence rate than low levels of K fertiliser inputs (Fig. 6b). Different fertiliser application strategies affected the crop emergence rate (*F*_7,35_ = 2.42, *p* < 0.05). The application of SCF and OIF decreased the proportion of potato emergence (Fig. 6c). The lowest plant emergence rate was associated with the input of the soil conservation fertiliser (SCF) at 300 kg ha^−1^.

### Average canopy cover

**Fig. 7:**
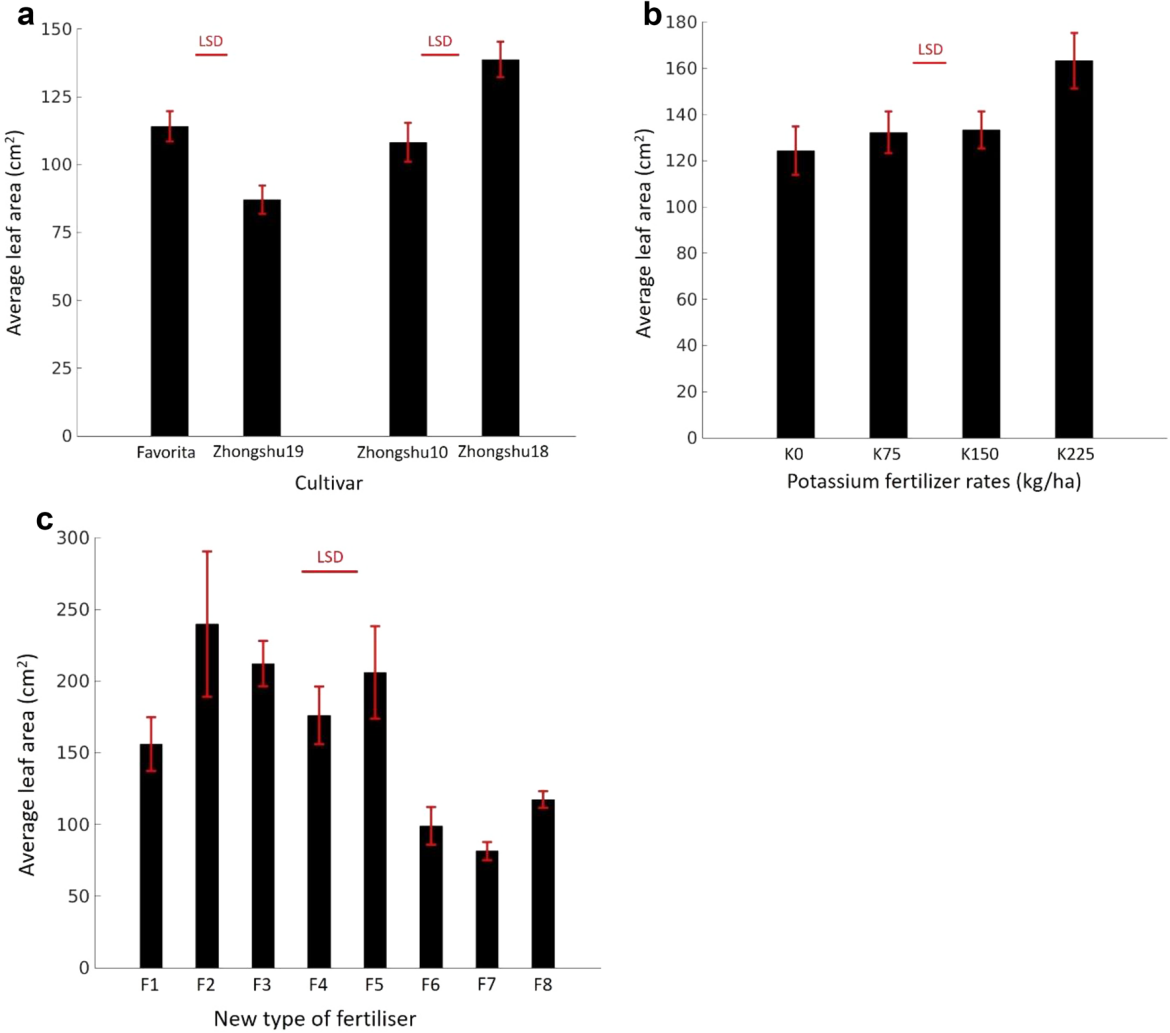
Estimated average canopy cover by the image analysis software for: **a** multiple cultivars (Experiment 1); **b** different input levels of K fertiliser (Experiment 2); and **c** different organic–inorganic compound fertiliser applications (Experiment 3). *LSD* least significant difference

Similar to the emergence rate, the cultivars differed in average canopy cover. ‘Favorita’ and ‘Zhongshu 18’ showed larger (*F*_1,14_ = 10.93, *p* < 0.01 and *F*_1,14_ = 11.48, *p* < 0.005) average canopy cover than ‘Zhongshu 19’ and ‘Zhongshu 10’, respectively (Fig. 7a). There was no significant difference among the three lower levels (0, 75 and 150 kg ha^−1^) of K fertiliser inputs. However, when the input increased to 225 kg ha^−1^, the average canopy cover increased significantly (Fig. 7b). Adding SCF and OIF fertilisers to the base fertiliser decreased potato emergence rate but significantly increased their average canopy cover (Fig. 7c). On the contrary, the CM fertiliser improved potato emergence rate, but reduced their average canopy cover.

### Variability in plant development

**Fig. 8:**
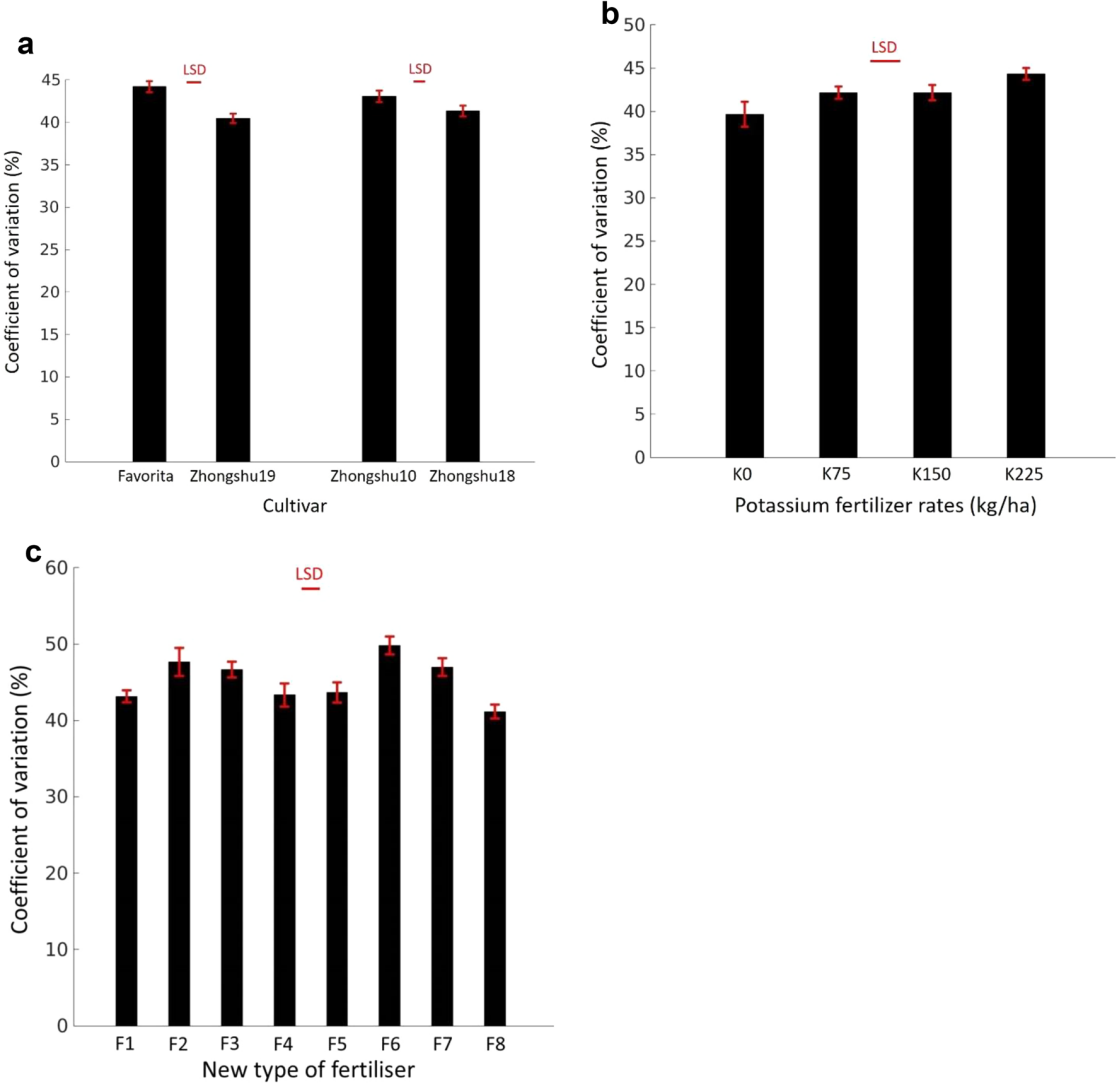
Average coefficient of variation of canopy cover per plant as estimated by the image analysis software for **a** multiple cultivars (Experiment 1), **b** different input levels of K fertiliser (Experiment 2), and **c** different organic–inorganic compound fertiliser applications (Experiment 3). *LSD* least significant difference

Figure 8 shows the average values of coefficient of variations of canopy cover per plant for all three experiments. In the cultivar experiment, two early maturing cultivars had higher CVs in canopy cover (hence lower uniformity) (*F*_1,14_ = 19.39, *p* < 0.001 and *F*_1,14_ = 3.93, *p* < 0.05) than the two late maturing cultivars (Fig. 8a). In the K experiment, CVs appeared to increase with increasing K input; however only the difference between two input levels (0 and 225 kg ha^−1^) was statistically significant (*F*_1,10_ = 6.90, *p* < 0.05) (Fig. 8b). For the compound fertiliser experiment, fertiliser treatments significantly (*F*_7,35_ = 8.76, *p* < 0.001) affected the variability in average canopy cover, with the best uniformity achieved by the lowest input of compound fertilisers (Fig. 8c).

## Discussion

UAV-based image analysis has become a powerful tool for high-throughput plant phenotyping at both field and plot levels in recent years. For crop emergence assessment, this approach has proven to be more suitable than other remote sensing techniques, such as airborne and satellite remote sensing, due to its flexibility, high spatial and temporal resolution. Although several studies have applied remote sensing for crop emergence evaluation, only one recent study [24] was focused on potato crop. In comparison to the study of Sankaran et al. [24], an RGB camera rather than a multispectral camera was equipped onto a low-altitude UAV in this study, indicating that cost of the imaging system can be reduced.

The excess green index with Otsu automatic thresholding method showed better performance than the ExG-ExR method. Because of the large variation of illumination across the experiment field, the Otsu thresholding was applied individually to each selected ROI in order to minimize segmentation errors. This method will be further validated under more illumination conditions and a white reference should be used in the future to make the images taken at different time points more comparable. A few of weeds were observed within the experiment field. These pixels might have been misclassified as potato plants due to their green colour. Weed mapping has been investigated in early-season maize fields by UAV multispectral imaging [40, 41], so, in future study, a weed identification method could also be incorporated to improve the accuracy of potato plant recognition. In the present study, canopy area data derived from vegetation images with manual segmentation were used as the ground truth for validating the image analysis method. In future studies, ground-based RGB images will be taken to estimate canopy cover to improve validate accuracy.

A Random Forest classifier has been implemented successfully to count potato plants with canopy overlaps in this study. Compared with other machine learning methods applied, such as ANN [42] and SVM [25], the Random Forest method can evaluate the importance of individual variable. Three most important morphological features for counting rapeseed seedling stand were identified as length–width ratio, density and perimeter [22]. The present study also indicated that the perimeter of plant objects is a highly important feature, which was directly related to the size of the plant object. However, the length–width ratio was not significant in this study due to the irregular shape of small potato canopies. The canopy area is less important due to the large variation in the canopy size, ranging from the early to well-developed stage, and overlaps between small plants. Not surprisingly, the solidity is the least important variable for plant counting because all plant objects with connected contours showed similar high solidity. The accuracy of plant counting is essential for a reliable assessment of average canopy cover and emergence uniformity. More training data with more morphological traits can potentially further improve the classification performance. The performance of the present prediction model should be further validated in future study, particularly for situations with many potato plants in a given plant object. In this study, the maximum number of plants within a connected contour was 8. Larger plant objects should be incorporated and trained to improve the robustness of the model in future experiments. Attention should also be paid to the UAV flight altitude and imaging sensor resolution as they could affect morphological feature values of plant objects. It was noticed that some tiny canopies were classified as background due to the resolution, so in future studies, UAV could fly at lower altitude or equipped with higher resolution camera. In order to improve the repeatability, a reference with known size should be placed in the field for spatial calibration of UAV images.

The present image analysis of the effect of cultivar, K fertiliser input and different organic–inorganic compound fertiliser combination applications was consistent with previous knowledge except the higher emergence of ‘Zhongshu 18’ cultivar than ‘Zhongshu 10’ cultivar, which indicated that the plant emergence rate was not necessarily higher for early maturing cultivars than for late maturing cultivars. After the dormancy period, the emergence of early maturing cultivars is always higher than late maturing cultivars under the same sprouting conditions. But cultivars with good sprouting at the time of sowing also tend to emerge early [43]. In order to ensure similar sprouting in the present study, each cultivar was moved from underground storage to greenhouse at different times aiming to achieve similar degrees of sprouting among different cultivars. ‘Zhongshu 18’ is a medium-late maturing cultivar with a dormancy period of around 45 to 60 days. It was moved to greenhouse on 15th April 2018, 7 and 13 days earlier than ‘Zhongshu 19’, and ‘Favorita’/’Zhongshu 10’, respectively. In the experiment, ‘Zhongshu 18’ showed better emergence than ‘Zhongshu 10’, which is probably caused by a longer sprouting time.

China’s potato planting area accounts for around 28% of the world’s potato production but the application of N fertiliser far exceed the international standard, resulting in serious resource waste and environmental pollution. With further development, the proposed UAV based image analysis can be applied as a high throughput phenotyping method in practice for monitoring potato crop development at the emergence stage, and greatly facilitate research for optimizing fertiliser management and improving emergence consistency.

## Conclusions

In this study, a UAV based image analysis software was developed for potato crop emergence assessment. High correlation was shown in plant emergence between the image analysis and manual counting. Compared with traditional manual assessment, this new approach can reduce the amount of time required to estimate crop emergence, average canopy cover and crop emergence uniformity. This software is highly adaptable and high throughput, and will greatly accelerate crop phenotyping in future potato agronomy and breeding research.

## References

[1] Zhang W, Liu X, Wang Q, Zhang H, Li M, Song B (2018). Effects of potassium fertilization on potato starch physicochemical properties. Int J Biol Macromol.

[2] Selladurai R, Purakayastha TJ (2015). Effect of humic acid multinutrient fertilizers on yield and nutrient use efficiency of potato. J Plant Nutr.

[3] Liang SM, Ren C, Wang PJ, Wang XT, Li YS, Xu FH (2018). Improvements of emergence and tuber yield of potato in a seasonal spring arid region using plastic film mulching only on the ridge. Field Crops Res.

[4] Spitters C J T, Schapendonk A H C. Evaluation of breeding strategies for drought tolerance in potato by means of crop growth simulation. In: Genetic aspects of plant mineral nutrition; 1990. p. 151–61.

[5] Ciuberkis S, Bernotas S, Raudonius S, Felix J (2007). Effect of weed emergence time and intervals of weed and crop competition on potato yield. Weed Technol.

[6] Moran MS, Inoue Y, Barnes EM (1997). Opportunities and limitations for image-based remote sensing in precision crop management. Remote Sens Environ.

[7] Van Loon CD (1981). The effect of water stress on potato growth, development, and yield. Am Potato J.

[8] Dyson PW, Watson DJ (1971). An analysis of the effects of nutrient supply on the growth of potato crops. Ann Appl Biol.

[9] Duan T, Zheng B, Guo W, Ninomiya S, Guo Y, Chapman SC (2017). Comparison of ground cover estimates from experiment plots in cotton, sorghum and sugarcane based on images and ortho-mosaics captured by UAV. Funct Plant Biol.

[10] Holman F, Riche A, Michalski A, Castle M, Wooster M, Hawkesford M (2016). High throughput field phenotyping of wheat plant height and growth rate in field plot trials using UAV based remote sensing. Remote Sens.

[11] Sankaran S, Khot LR, Espinoza CZ, Jarolmasjed S, Sathuvalli VR, Vandemark GJ (2015). Low-altitude, high-resolution aerial imaging systems for row and field crop phenotyping: a review. Eur J Agron.

[12] Zarco-Tejada PJ, González-Dugo V, Berni JAJ (2012). Fluorescence, temperature and narrow-band indices acquired from a UAV platform for water stress detection using a micro-hyperspectral imager and a thermal camera. Remote Sens Environ.

[13] Arnold T, De Biasio M, Fritz A, Leitner R (2010). UAV-based multispectral environmental monitoring. Sensors.

[14] Yuan Y, Hu X (2016). Random forest and objected-based classification for forest pest extraction from UAV aerial imagery. ISPRS Int Arch Photogramm Remote Sens Spat Inf Sci.

[15] Smigaj M, Gaulton R, Barr SL, Suárez JC (2015). UAV-borne thermal imaging for forest health monitoring: detection of disease-induced canopy temperature increase. ISPRS Int Arch Photogramm Remote Sens Spat Inf Sci.

[16] Peña JM, Torres-Sánchez J, de Castro AI, Kelly M, López-Granados F (2013). Weed mapping in early-season maize fields using object-based analysis of unmanned aerial vehicle (UAV) images. PLoS ONE.

[17] McNeil BE, Pisek J, Lepisk H, Flamenco EA (2016). Measuring leaf angle distribution in broadleaf canopies using UAVs. Agric For Meteorol.

[18] Schirrmann M, Giebel A, Gleiniger F, Pflanz M, Lentschke J, Dammer K-H (2016). Monitoring agronomic parameters of winter wheat crops with low-cost UAV imagery. Remote Sens.

[19] Duan S-B, Li Z-L, Wu H, Tang B-H, Ma L, Zhao E (2014). Inversion of the PROSAIL model to estimate leaf area index of maize, potato, and sunflower fields from unmanned aerial vehicle hyperspectral data. Int J Appl Earth Obs Geoinf.

[20] Maresma Á, Ariza M, Martínez E, Lloveras J, Martínez-Casasnovas J (2016). Analysis of vegetation indices to determine nitrogen application and yield prediction in maize (*Zea mays* L.) from a standard UAV service. Remote Sens.

[21] Zhou X, Zheng HB, Xu XQ, He JY, Ge XK, Yao X (2017). Predicting grain yield in rice using multi-temporal vegetation indices from UAV-based multispectral and digital imagery. ISPRS J Photogramm Remote Sens.

[22] Zhao B, Zhang J, Yang C, Zhou G, Ding Y, Shi Y (2018). Rapeseed seedling stand counting and seeding performance evaluation at two early growth stages based on unmanned aerial vehicle imagery. Front Plant Sci.

[23] Makanza R, Zaman-Allah M, Cairns J, Magorokosho C, Tarekegne A, Olsen M (2018). High-throughput phenotyping of canopy cover and senescence in maize field trials using aerial digital canopy imaging. Remote Sens.

[24] Sankaran S, Quirós JJ, Richard Knowles N, Knowles LO (2017). High-resolution aerial imaging based estimation of crop emergence in potatoes. Am J Potato Res.

[25] Jin X, Liu S, Baret F, Hemerlé M, Comar A (2017). Estimates of plant density of wheat crops at emergence from very low altitude UAV imagery. Remote Sens Environ.

[26] Meyer GE, Neto JC (2008). Verification of color vegetation indices for automated crop imaging applications. Comput Electron Agric.

[27] Oualline S (2003). Practical C++ programming.

[28] Yu Z, Cao Z, Wu X, Bai X, Qin Y, Zhuo W (2013). Automatic image-based detection technology for two critical growth stages of maize: emergence and three-leaf stage. Agric For Meteorol.

[29] Minervini M, Abdelsamea MM, Tsaftaris SA (2014). Image-based plant phenotyping with incremental learning and active contours. Ecol Inform.

[30] Sadeghi-Tehran P, Virlet N, Sabermanesh K, Hawkesford MJ (2017). Multi-feature machine learning model for automatic segmentation of green fractional vegetation cover for high-throughput field phenotyping. Plant Methods.

[31] Woebbecke DM, Woebbecke DM, Meyer GE, Von Bargen K, Mortensen DA (1995). Color indices for weed identification under various soil, residue, and lighting conditions. Trans ASAE.

[32] Otsu N (1979). A threshold selection method from gray-level histograms. IEEE Trans Syst Man Cybern.

[33] Ponti MP (2013). Segmentation of low-cost remote sensing images combining vegetation indices and mean shift. IEEE Geosci Remote Sens Lett.

[34] Pal M (2005). Random forest classifier for remote sensing classification. Int J Remote Sens.

[35] Breiman L (2017). Classification and regression trees.

[36] Grimm R, Behrens T, Märker M, Elsenbeer H (2008). Soil organic carbon concentrations and stocks on Barro Colorado Island—digital soil mapping using Random Forests analysis. Geoderma.

[37] Gray KR, Aljabar P, Heckemann RA, Hammers A, Rueckert D (2013). Alzheimer’s disease neuroimaging initiative. Random forest-based similarity measures for multi-modal classification of Alzheimer’s disease. Neuroimage.

[38] Calera A, Martínez C, Melia J (2001). A procedure for obtaining green plant cover: relation to NDVI in a case study for barley. Int J Remote Sens.

[39] Tallarida RJ, Murray RB. Least significant difference test. Manual of pharmacologic calculations; 1987. p. 128–30.

[40] López-Granados F, Torres-Sánchez J, Serrano-Pérez A, de Castro AI, Mesas-Carrascosa FJ, Peña JM (2016). Early season weed mapping in sunflower using UAV technology: variability of herbicide treatment maps against weed thresholds. Precis Agric.

[41] Peña JM, Torres-sánchez J, de Castro AI, Kelly M, López-Granados F (2013). Weed mapping in early-season maize fields using object-based analysis of unmanned aerial vehicle (UAV) images. PloS ONE.

[42] Liu S, Baret F, Andrieu B, Burger P, Hemmerlé M (2017). Estimation of wheat plant density at early stages using high resolution imagery. Front Plant Sci.

[43] Kinyua ZM, Schulte-Geldermann E, Namugga P, Ochieng-Obura B, Tindimubona S, Bararyenya A, et al. Adaptation and improvement of the seed-plot technique in smallholder potato production. In: Low J, Nyongesa M, Quinn S, Parker M (eds) Potato and sweetpotato in Africa: transforming the value chains for food and nutrition security. CABI; 2015. p. 218–25.

